# Improving the recall of biomedical named entity recognition with label re-correction and knowledge distillation

**DOI:** 10.1186/s12859-021-04200-w

**Published:** 2021-06-02

**Authors:** Huiwei Zhou, Zhe Liu, Chengkun Lang, Yibin Xu, Yingyu Lin, Junjie Hou

**Affiliations:** 1 School of Computer Science and Technology, Ganjingzi District, Dalian University of Technology, Address Chuangxinyuan Building, No.2 Linggong Road, Dalian, 116024 Liaoning China; 2 School of Foreign Languages, Ganjingzi District, Dalian University of Technology, Address Chuangxinyuan Building, No.2 Linggong Road, Dalian, 116024 Liaoning China; 3 School of Business, Panjin Campus of Dalian University of Technology, No. 2 Dagong Road, Liaodongwan New District, PanJin, 124221 Liaoning China

**Keywords:** Biomedical named entity recognition, Knowledge distillation, Label re-correction

## Abstract

**Background:**

Biomedical named entity recognition is one of the most essential tasks in biomedical information extraction. Previous studies suffer from inadequate annotated datasets, especially the limited knowledge contained in them.

**Methods:**

To remedy the above issue, we propose a novel Biomedical Named Entity Recognition (BioNER) framework with label re-correction and knowledge distillation strategies, which could not only create large and high-quality datasets but also obtain a high-performance recognition model. Our framework is inspired by two points: (1) named entity recognition should be considered from the perspective of both coverage and accuracy; (2) trustable annotations should be yielded by iterative correction. Firstly, for coverage, we annotate chemical and disease entities in a large-scale unlabeled dataset by PubTator to generate a weakly labeled dataset. For accuracy, we then filter it by utilizing multiple knowledge bases to generate another weakly labeled dataset. Next, the two datasets are revised by a label re-correction strategy to construct two high-quality datasets, which are used to train two recognition models, respectively. Finally, we compress the knowledge in the two models into a single recognition model with knowledge distillation.

**Results:**

Experiments on the BioCreative V chemical-disease relation corpus and NCBI Disease corpus show that knowledge from large-scale datasets significantly improves the performance of BioNER, especially the recall of it, leading to new state-of-the-art results.

**Conclusions:**

We propose a framework with label re-correction and knowledge distillation strategies. Comparison results show that the two perspectives of knowledge in the two re-corrected datasets respectively are complementary and both effective for BioNER.

## Introduction

Biomedical Named Entity Recognition (BioNER) is a fundamental step for downstream biomedical natural language processing tasks. BioNER is a great challenge due to the following reasons: various ways of naming biomedical entities, ambiguities caused by the frequent occurrences of abbreviations, and new entities constantly and rapidly reported in scientific publications [1]. To promote the performance of BioNER, many challenging BioNER tasks have been proposed, such as chemical and disease named entity recognition in the BioCreative V chemical-disease relation (CDR) task [2] and disease named entity recognition in the NCBI Disease task [3].

Recent Named Entity Recognition (NER) studies employ neural network models to generate quality features [4, 5]. However, neural network models require large-scale datasets to train millions of parameters. It is too expensive and time-consuming to manually annotate large-scale datasets.

This motivates some researchers to automatically create large-scale annotation datasets with semi-structured resources and semi-supervised methods [6, 7]. They generate named entity annotations by leveraging the link structure of Wikipedia.

Inevitably, these methods generate many false labels during the annotation process. Zhu et al. [8] design a neural correction model trained with a small human-annotated NER dataset to correct false labels. They illustrate that correction process could greatly improve the quality of the annotation dataset. Nevertheless, noisy labels still exist and cannot be further reduced by their method.

Bagherinezhad et al. [9] propose an iterative process called Label Refinery to reduce false labels caused by crop-level augmentation, and observe that labels improve iteratively even when the same architecture model is used to refine the dataset multiple times.

In biomedical domain, there is no large-scale semi-structured dataset like Wikipedia. Instead, many large-scale structured knowledge bases are constructed, such as CTDbase [10], MeSH [11] and RGD [12]. These repositories link PubMed identifiers (PMIDs) with entity identifiers (IDs), such as <PMID: 6893628, disease ID: D010264> from MeSH. How to make use of these resources for BioNER is more challenging, and becomes an urgent demand. Wei et al. [13] first collect mentions from structured knowledge bases, and then correlate them with the text mined span from a name entity recognition and link tool PubTator [14] for mention disambiguation.

In addition, it has been long observed that combining the predictions of multiple networks usually exceeds the performance of an individual network. Unfortunately, the space to store multiple networks and the time to execute them at prediction time prohibit their use, especially when the individual models are large scale neural networks. Recently, a promising ensemble method, knowledge distillation [15–17], is raised to overcome this problem. It could distill diverse knowledge from different trained (teacher) models into a single (student) model.

Considering coverage and accuracy of NER, we construct two datasets. As for coverage, we automatically annotate the spans of chemical and disease mentions in a large-scale unlabeled dataset by PubTator to construct a weakly labeled dataset. And as for accuracy, multiple large-scale structured knowledge bases (i.e. CTDbase, MeSH, RGD) are utilized to filter out the mentions if their IDs are not contained in the current PMID. In this way, we construct two large-scale weakly labeled datasets.

Next, we propose a novel label re-correction strategy to improve the recall without significantly introducing noise in the weakly labeled datasets iteratively, and obtain two high-quality complementary datasets. They are used to train two BioNER models, respectively.

Finally, to integrate diverse knowledge in the two models and save time and space, we utilize them as teachers to teach a distilled student model with knowledge distillation.

In summary, we mainly make the following contributions:

- We construct two weakly labeled datasets considering from coverage and accuracy respectively by utilizing multiple knowledge bases and PubTator.
- We propose a novel label re-correction strategy for iteratively improving the recall without significantly introducing noise in the weakly labeled datasets, and obtain two high-quality datasets.
- We introduce knowledge distillation to compress the recognition models trained on the two datasets into a single recognition model. Experimental results show that our model yields state-of-the-art results on the CDR and NCBI Disease corpus.

## Related work

Most existing approaches treat BioNER as a sequence tagging problem. Recently, various neural network architectures have been proposed for BioNER with word and character embeddings, among which bidirectional long short-term memory with conditional random field (BiLSTM-CRF) model exhibits promising results [5].

Besides word and character features, linguistic features and domain resource features [1, 18, 19] are also used to enrich the information of each token. These approaches heavily rely on quality and quantity of the labeled corpora. However, such BioNER resources of each entity type are scarce.

To address this problem, datasets of different types of entities are used to augment resources for knowledge transfer by multi-task learning [20–22]. However, combining several limited datasets of different tasks could hardly meet the needs of large-scale training parameters, and the relatedness among tasks usually limits NER performance.

A recent trend in transfer learning is to take advantage of unlimited amount of unlabeled datasets by unsupervised pre-training. BERT is designed to pre-train language representations with large-scale unlabeled datasets, which has been proved effective for improving many natural language processing tasks [23]. Lee et al. [24] pre-train BioBERT on general and biomedical domain corpora, and illustrate that it achieves better performance than BERT on BioNER tasks.

##  Methods

**Fig. 1:**
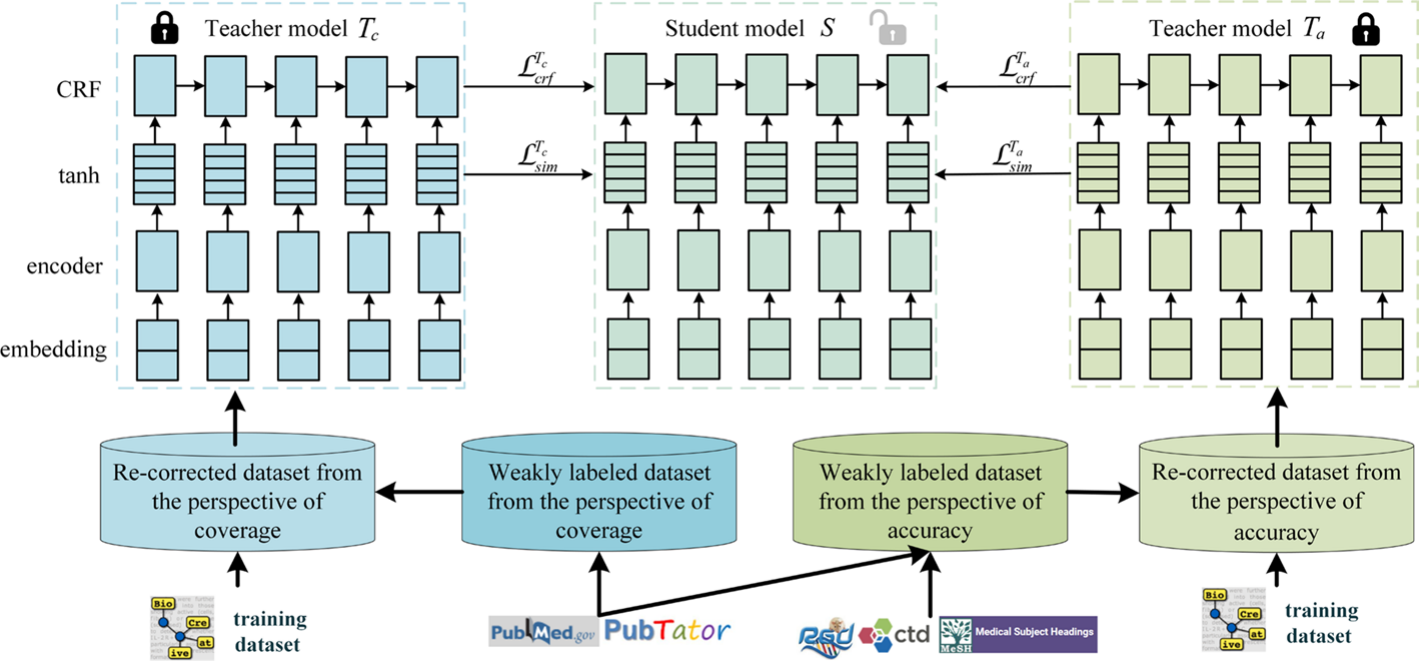
The framework of our BioNER with label re-correction and knowledge distillation

In this section, we introduce our neural network-based BioNER framework with label re-correction and knowledge distillation strategies, as shown in Fig. 1. Firstly, two large-scale weakly labeled datasets are constructed with Pubtator and knowledge bases (“Weakly labeled dataset construction” section). Then we apply BiLSTM-CRF or BioBERT-CRF as the basic model (“Basic model” section) and correct noisy labels iteratively with label re-correction strategy (“Label re-correction strategy” section). Finally, we utilize knowledge distillation to compress the knowledge in two teacher models trained on the two re-corrected datasets into a student model (“Knowledge distillation” section).

###  Weakly labeled dataset construction

**Fig. 2:**
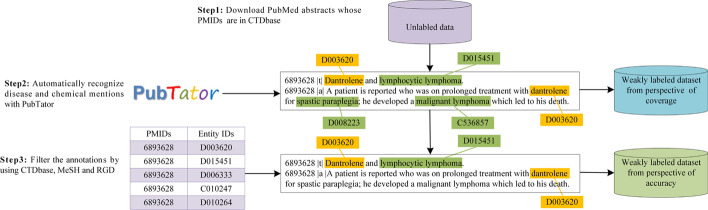
Illustration of the dataset generation pipeline from the perspectives of coverage and accuracy. The chemical and disease mentions are highlighted in yellow and green, respectively.

Inspired by Wei et al. [13], we use both the records in knowledge bases and the text mined span from PubTator for dataset generation. Two large-scale weakly labeled datasets are automatically constructed for coverage and accuracy, respectively. As shown in Fig. 2, the pipeline used to create two datasets is illustrated in the following steps:

Step 1: Download PubMed abstracts whose PMIDs are in CTDbase since these abstracts contain both chemical and disease entities. CTDbase associates PMIDs with pairs of chemical-disease relations, such as < PMID: 6893628, Chemical ID: D003620, Disease ID: D015451 > . However, none of the repositories provides the location of the mentions.

Step 2: Automatically recognize chemical and disease mentions with PubTator to obtain the first weakly labeled dataset. PubTator provides spans of mentions, which are automatically extracted by machine learning-based taggers. These taggers were previously evaluated and achieved 80–90% of *F*-score in recognition and normalization. From the perspective of coverage, we do not filter any mentions.

Step 3: Filter the spans whose entity IDs are not associated with the current PMID by using the repositories, i.e. CTDbase, MeSH, RGD. Obviously, many false positives exist in the first dataset. From the perspective of accuracy, only the spans that are matched with the repository records are remained. For example, the span of “spastic paraplegia” with entity ID D008223 and the span of “malignant lymphoma” with entity ID C536857 recognized by PubTator in Fig. 2 are filtered because they are not recorded in PMID 6893628 in repositories. Note that, CTDbase only associates PMIDs with pairs of chemical-disease relations. Therefore, MeSH and RGD are employed to complement records.

In this way, two Chemical and Disease Weakly labeled datasets for Coverage and Accuracy are created called CDWC and CDWA with same abstracts but different annotations. The statistics of the two datasets are listed in the first two rows of Table 1.

**Table 1:** Various statistics of the datasets

Dataset		#Abstract	#Chemical	#Disease	$Chemical	$Disease
Weakly labeled	CDWC	70,026	706,593	514,964	34,696	58,985
	CDWA	70,026	503,700	283,293	17,939	24,600
	CDRC (BiLSTM-CRF)	70,026	770,159	541,235	40,135	38,715
	CDRA (BiLSTM-CRF)	70,026	781,039	532,198	38,858	42,420
	CDRC (BioBERT-CRF)	70,026	795,096	557,434	50,018	52,447
	CDRA (BioBERT-CRF)	70,026	812,516	542,353	51,458	47,687
	DRC (BiLSTM-CRF)	70,026	–	469,849	–	69,567
	DRA (BiLSTM-CRF)	70,026	–	473,728	–	69,342
	DRC (BioBERT-CRF)	70,026	–	546,515	–	83,436
	DRA (BioBERT-CRF)	70,026	–	487,636	–	66,582
Human annotated	CDR training data	500	5203	4182	991	1384
	CDR development data	500	5347	4244	976	1254
	CDR test data	500	5385	4424	1239	1474
	NCBI disease training data	593	–	5145	–	1495
	NCBI disease development data	100	–	787	–	334
	NCBI disease test data	100	–	960	–	382

^#^Abstract: the number of abstracts

^#^Chemical: the number of chemical mentions

^#^Disease: the number of disease mentions

^$^Chemical: the number of unique chemical mentions

^$^Disease: the number of unique disease mentions

###  Basic model

We use BiLSTM-CRF or BioBERT-CRF model as our basic model, which has four layers as shown in Fig. 1.

In the embedding layer, for BiLSTM-CRF, a sentence ${\mathbf{w}} = < w_{1} ,w_{2} , \ldots ,w_{n} >$ is represented as ${\mathbf{X}} = < {\mathbf{x}}_{1} ,{\mathbf{x}}_{2} , \ldots ,{\mathbf{x}}_{n} >$, where ${\mathbf{x}}_{i}$ is the concatenation of 100-dimension word embedding pretrained on the PubMed articles provided by Wei et al. [14] and character embedding learned by a character-level convolutional neural network [4]. For BioBERT-CRF, we use the tokenization and embedding layer provided by Lee et al. [24]

In the encoder layer, for BiLSTM-CRF, ${\mathbf{X}}$ is fed to a BiLSTM layer to obtain the hidden representation of each token by concatenating its forward and backward context representations. For BioBERT-CRF, ${\mathbf{X}}$ is fed to BioBERT to catch the context information.

The tanh layer consists of two linear transformations with a Tanh activation in between. It is used to predict confidence scores ${\mathbf{P}} = < P_{1} ,P_{2} ,...,P_{n} > \in R^{k \times n}$ for all tokens, where $k$ is the number of distinct labels.

Finally, a CRF layer is applied to decode the best tag path in all possible tag paths. The score of ${\mathbf{X}}$ with a sequence of labels ${\mathbf{y}} = < y_{1} ,y_{2} ,...,y_{n} >$ is defined as the sum of transition scores and confidence scores:

1 $$s({\mathbf{X}},{\mathbf{y}}) = \sum\limits_{i = 1}^{n} {(T_{{y_{i - 1} ,y_{i} }} + P_{{i,y_{i} }} )}$$

where $T_{i,j}$ represents the transition score from the *i*-th tag to the *j*-th tag.

During the training phase, the loss of the basic model is defined by:

2 $${L}_{crf} = - \log \frac{{e^{{s({\mathbf{X}},{\mathbf{y}})}} }}{{\sum\limits_{{{\mathbf{y}}^{\prime} \in {\mathbf{Y}}_{{\mathbf{X}}} }} {e^{{s({\mathbf{X}},{\mathbf{y}}^{\prime})}} } }}$$

where ${\mathbf{Y}}_{{\mathbf{X}}}$ are all possible tag paths.

At inference time, Viterbi algorithm [25] is adopted to search for the label sequence with the highest conditional probability.

###  Label re-correction strategy

Inevitably, many false negative annotations exist in CDWC and CDWA. In this paper, we propose a novel label re-correction strategy to improve the recall without significantly introducing noise in the weakly labeled datasets by leveraging a small manually-annotated dataset, i.e. CDR or NCBI Disease. Here BiLSTM-CRF or BioBERT-CRF is used as our correction model.

There are two intuitions behind our label re-correction strategy: (1) the annotations in training dataset can help us learn how to generate annotations in the large-scale dataset; (2) the iterative procedure to update labels can improve both the dataset and the trained correction models.

Given training data ***T***, development data ***D*** and a large-scale dataset ***L***, the process of label re-correction is defined as follows: we firstly train a new correction model *C* on ***L***; then we transfer the model *C* to ***T*** through fine-tuning *C* on ***T***; finally, the correction model *C* is used to correct the label sequences in ***L***. We put the sentence through the correction model *C* and rewriting the old tags with the new output of the correction model *C*. We repeat such a correct procedure until the *F*-score on development data ***D*** does not increase.

CDR corpus contains 1500 PubMed abstracts: 500 each for training, development and test set, as shown in Table 1. Following Luo et al. [1], the original training set and development set are merged, and we randomly select 10% of them as development data ***D*** and the rest is training data ***T***. Two weakly labeled datasets CDWA and CDWC are **R**e-corrected to obtain two corresponding high-quality datasets called CDRA and CDRC, respectively.

For NCBI Disease, as shown in Table 1, we directly use development data as ***D*** and training data as ***T***. Two weakly labeled datasets CDWA and CDWC are **R**e-corrected to obtain two corresponding high-quality datasets called DRA and DRC, respectively.

The statistics of the Re-corrected datasets are listed in Table 1. We can see that the number and coverage of chemical and disease annotations in most of the re-corrected datasets are both larger than those in original weakly labeled datasets. We believe that label re-correction strategy could effectively correct the false-negative entity labels.

###  Knowledge distillation

Two re-corrected datasets aim to annotate chemical and disease entities from the perspectives of coverage and accuracy, respectively. We use them to train two recognition models $T_{j} ,j \in \{ {\text{c,a}}\}$, which are complementary.

We calculate the label similarity of each abstract predicted by two recognition models over large-scale dataset as follows:

3 $$label\_similarity = \frac{\# same}{{\# total}}$$

where #same is the number of the words which have the same labels predicted by two recognition models, and #total is the number of the words in an abstract. We adopt the IOB tagging scheme, in which I stands for Inside, O stands for Outside, B stands for Beginning.

**Fig. 3:**
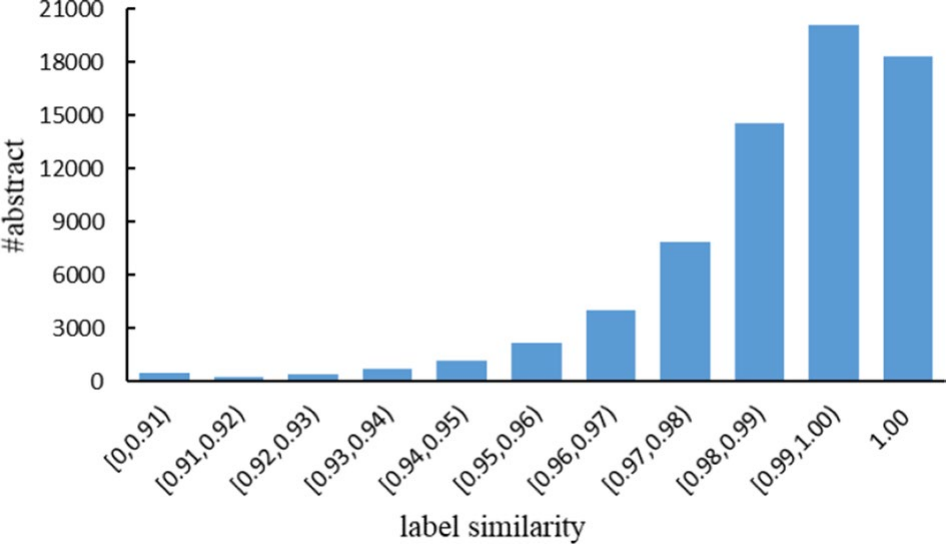
Label similarity distribution over the large-scale dataset between the predictions of the two teacher models. Each bar represents the number of the abstracts with the probabilities of label similarity in the similarity interval.

The label similarity distribution is shown in Fig. 3. From this figure, we can see that though most of predicted labels are same, there are still a lot of differences between the two models. For the abstracts with label similarity less than 1, even the label similarity is high, there are still many different entities predicted by two recognition models. It is because that most of the same labels are O labels. For BioNER, there are inevitable many O labels in an abstract. The distribution means that each of the two recognition models still have its own knowledge. It is natural to combine them to get a better model.

We distill the knowledge from the two recognition models (teacher) and transfer it to a new recognition model (student). The structures of teachers and student could be identical or different. In this paper, the teachers $T_{j} ,j \in \{ {\text{c,a}}\}$ and the student $S$ are based on the same architecture. In this way, at inference time, comparing with using two teacher models, using the student model only spends half time and memory space. Though the training of student model takes more time.

The label sequences (hard labels) ${\mathbf{y}}$ and the confidence scores ${\mathbf{P}}$ (used to calculate soft labels) predicted by the two teacher models are both used to teach the student model. Once the teacher models are trained, their parameters are frozen during the student model training.

With the hard labels, the student model is trained by minimizing the loss of ${L}_{crf}^{{T_{j} }}$ in Eq. (2) based on ${\mathbf{y}}^{{T_{j} }}$ predicted by the teacher model $T_{j}$.

With the soft labels, the student model is trained by minimizing the similarity distance between soft labels of the reference teacher and the estimated student. The similarity metric can be formulated as follows:

4 $${L}_{sim}^{{T_{j} }} = \sum\limits_{i = 1}^{n} {d(q_{i}^{{T_{j} }} ,q_{i}^{S} )}$$

where $q_{i}^{{T_{j} }}$ and $q_{i}^{S}$ are soft labels generated by the teacher and the student, respectively, $d$ is referred to as a distance function. In this work, we investigate three distance metrics as follows:

- $l_{1}$** distance**: We apply a softmax layer on confidence scores $P_{i}^{{T_{j} }}$ and $P_{i}^{S}$ of each token to get the corresponding soft labels $q_{i}^{{T_{j} }}$ and $q_{i}^{S}$. $l_{1}$ distance is the absolute differences between the soft labels:
  5 $${L}_{{l_{1} \_sim}}^{{T_{j} }} = \, \frac{1}{n}\sum\limits_{i = 1}^{n} {{|}q_{i}^{{T_{j} }} - q_{i}^{S} |}$$
- $l_{2}$ **distance**: Here, the soft labels are the same as those used in $l_{1}$ distance. $l_{2}$ distance is the straight-line distance in euclidean space between the soft labels:
  6 $${L}_{{l_{2} \_sim}}^{{T_{j} }} = \, \frac{1}{n}\sum\limits_{i = 1}^{n} {{||}q_{i}^{{T_{j} }} - q_{i}^{S} ||_{2}^{2} }$$
- $l_{KD}$ **distance**: Following Hinton et al. [15], we use a softmax layer to convert $P_{i}^{{T_{j} }} /t$ and $P_{i}^{S} /t$ to soft labels $q_{i}^{{T_{j} }}$ and $q_{i}^{S}$, where $t$ is the temperature. Then the $l_{KD}$ distance is defined as the cross-entropy between the soft labels multiplied by $t^{2}$:
  7 $${L}_{KD\_sim}^{{T_{j} }} = \, \frac{{t^{2} }}{n}\sum\limits_{i = 1}^{n} {q_{i}^{{T_{j} }} \log q_{i}^{S} }$$

The final objective loss for the distilled model is the sum of the hard label losses and the soft label losses:

8 $${L} = \sum\limits_{{j \in \{ {\text{c,a}}\} }} {{L}_{sim}^{{T_{j} }} + {L}_{crf}^{{T_{j} }} }$$

##  Experiment and discussion

###  Experimental settings

#### Dataset and evaluation metrics

We evaluate the proposed framework on CDR and NCBI Disease test set. The evaluation is reported by official evaluation toolkit, which adopts the standard Precision (*P*), Recall (*R*) and *F*-score (*F*) to measure the performance.

#### Implementation details

Word2Vec [26] is employed to pretrain 100-dimension word embeddings on the PubMed articles provided by Wei et al. [14]. Other parameters are initialized randomly from uniform distributions. The dimension of character embeddings is 50. Mini-batch size is set to 32 and 4 for the model trained on the large-scale dataset and training dataset, respectively. RMSProp optimizer with learning rate 1e-3 is used to minimize the loss. All the experiments were conducted on GeForce GTX 1080 using PyTorch. The runtime of the different models is shown in Table 2.

**Table 2:** The runtime of the experiments

Models	Time (min)
Weakly labeled (BiLSTM-CRF)	320
Training (BiLSTM-CRF)	2
Distillation (BiLSTM-CRF)	625
Weakly labeled (BioBERT-CRF)	334
Training (BioBERT-CRF)	4
Distillation (BioBERT-CRF)	554

“Time” denotes training time for one epoch. “Weakly labeled” and “Training” are the training time of the model trained on the weakly labeled dataset and CDR training dataset, respectively. “Distillation” is the training time of knowledge distillation

###  Effects of label re-correction

We first investigate the effects of the label re-correction strategy. Since we do not have the gold labels of weakly labeled datasets, we use the performance on the CDR test set to show the quality of the re-corrected dataset. Table 3 shows the results of the BiLSTM-CRF model trained on the two weakly labeled datasets, respectively. For CDWC and CDWA, the label re-correction process is repeated multiple times before convergence.

**Table 3:** Comparison of BiLSTM-CRF model results trained on CDWC and CDWA with different re-correction times

Dataset	*P* (%)	*R* (%)	*F* (%)	Dataset	*P* (%)	*R* (%)	*F* (%)
CDR	91.42	83.59	87.86	CDR	91.42	83.59	87.86
CDR + CDWC	90.17	84.49	87.24	CDR + CDWA	94.02	71.02	80.92
CDWC	89.72	83.65	86.58	CDWA	94.75	67.27	78.68
CDWC^1^	89.84	89.32	89.58	CDWA^1^	90.16	88.94	89.55
CDWC^2^	90.00	89.35	89.67	CDWA^2^ (CDRA)	91.03	88.31	**89.65**
CDWC^3^(CDRC)	89.80	89.82	**89.81**	CDWA^3^	90.28	89.03	89.65
CDWC^4^	89.90	89.70	89.80				

The highest scores are highlighted in bold

All results are evaluated on the CDR test set. The first two lines are the baselines. For the last 5 lines, each dataset is constructed by the correction model trained with the dataset right above it. The superscript represents the re-correction times. That is, CDWC^1^ is the dataset constructed by the correction model trained on the CDWC. The third row datasets are the weakly labeled datasets without re-correction. What’s more, CDWC^3^ is CDRC, and CDWA^2^ is CDRA

Comparing the first two lines in the table, the model trained on the CDR training set perform better than the models trained on the combination of CDR training set and weakly labeled datasets. This proves that there are many false negative labels in weakly labeled datasets.

In addition, we can observe that although the first re-correction process significantly improves the *F*-score, especially the recall, correcting only once is not enough. As the label re-correction process is further performed iteratively, the labels of the two datasets improve gradually, and thereby benefit the correction models.

Afterwards, we also find that CDRA and CDRC have a positive effect on recall comparing with CDWA and CDWC, respectively. However, the precision of CDRA is lower than CDWA. The reason for the reduced precision is perhaps that each correction procedure pays close attention to the *F*-score rather than the property of dataset itself.

Finally, we can see that the results on CDWC datasets keep a relatively high recall, while those on CDWA datasets have a relatively high precision, which is in line with our original motivation.

### Different combinations of knowledge distillation

We further explore the effects of knowledge distillation on the CDR test set, which are summarized in Table 4. We investigate the influences of different combinations of hard label losses and soft label losses for knowledge distillation.

**Table 4:** Performance comparison of the distilled models trained with different combinations of losses

${L}_{crf}$	${L}_{crf}^{T}$	${L}_{KD\_sim}^{T}$	${L}_{{l_{1} \_sim}}^{T}$	${L}_{{l_{2} \_sim}}^{T}$	Adv	*F* (%)
✔						89.99
	✔					90.13
	✔	✔				90.16
	✔		✔			90.13
	✔			✔		**90.35**
	✔			✔	✔	90.16

The highest scores are highlighted in bold

Adv: the short for adversarial learning

The first row indicates the model trained on the combination of CDRA and CDRC. Comparing this row with others, it is observed that without knowledge distillation, the performance drops, which demonstrate the effectiveness of knowledge distillation. The second row indicates the model only use hard label losses. Comparing this row with the rows using both hard label losses and soft label losses, to our surprise, it is observed that using single hard labels can achieve competitive performance with both soft labels and hard labels. It is probably because the training dataset is so large that hard labels could contain most of the information encoded in soft labels.

Besides, adversarial learning is commonly used in knowledge distillation. We also introduce adversarial learning into our model as Shen et al. [17] do (last row in Table 4). Unfortunately, it does not work. The possible reason is that there exists some potential conflict of information between the two teachers. It is difficult to force the student to generate similar outputs to the two teachers’ at the same time.

###  Ablation study

To better understand the function of key components of our framework, we conduct some ablation studies on the CDR test set in Table 5.

**Table 5:** Ablation study results

Model	*P* (%)	*R* (%)	*F* (%)
Our best (BiLSTM-CRF)	90.71	**89.99**	**90.35**
*w/o label re-correction*	**91.34**	80.76	85.73**
*w/o CDRC*	90.48	89.14	89.81*
*w/o CDRA*	90.17	89.55	89.86**

The highest scores are highlighted in bold

w/o label re-correction: we train the teachers on the two weakly labeled datasets CDWC and CDWA rather than CDRC and CDRA

w/o CDRC: we train a single teacher without CDRC (i.e. only with CDRA)

w/o CDRA: we train a single teacher without CDRA (i.e. only with CDRC)

the marker * and ** represent *P *value < 0.05 and *P *value < 0.01, respectively, using pairwise t-test against our best (BiLSTM-CRF). Firstly, the formula of the pairwise t-test is defined as the sum of the differences of each pair divided by the square root of n times the sum of the differences squared minus the sum of the squared differences, overall n − 1. n is the number of pair. Then in this paper we use a two-tailed test in which the critical area of a distribution is two-sided and tests whether a sample is greater than or less than a certain range of values

####  Does label re-correction strategy really need to be applied to the weakly labeled datasets?

See the second row, instead of using re-corrected datasets CDRC and CDRA, we use weakly labeled datasets CDWC and CDWA to train the teachers. The recall of the distilled student model drops significantly. This proves the effectiveness of label re-correction, especially for reducing false negatives in the weakly labeled datasets.

#### Are both the datasets for coverage and accuracy beneficial?

See the last two rows, when we only use the dataset from one perspective, the performance of each student model drops but is still promising. This suggests that the datasets from two perspectives are complementary and both effective. It also proves the effectiveness of knowledge distillation.

### Main results

We compare our distilled recognition model with state-of-the-art methods on the BioCreative V CDR task and NCBI Disease task in Table 6. The BiLSTM-CRF and BioBERT-CRF model trained on the CDR and NCBI Disease training dataset are our baselines. These relevant models are divided into four groups. Except our model encoded with BioBERT and the method proposed by Lee et al. [24], all these methods are based on BiLSTM-CRF. To compare with other method in detail, the evaluation is performed on chemical type, disease type and both types.

**Table 6:** Comparison with some state-of-the-art methods

	Methods	CDR chemical ***F***(%)	CDR disease ***F***(%)	CDR both ***F***(%)	NCBI disease ***F***(%)
1	Habibi et al. [5]	91.05	83.49	87.63*	84.44
	Our baseline (BiLSTM-CRF)	91.42	83.59	87.86	83.96
	Our baseline (BioBERT-CRF)	93.69	86.19	90.31	87.47
2	Luo et al. [1]	92.57	–	–	–
	Dang et al. [18]	93.14	84.68	89.30*	84.41
3	Wang et al. [21]	–	–	88.78	86.14
	Yoon et al. [22]	92.74	82.61	88.15*	86.36
4	Lee et al. [24]	93.47	87.15	90.60*	89.71
	Our model (BiLSTM-CRF)	94.17	85.69	90.35	85.71
	Our model (BioBERT-CRF)	**95.22**	**87.34**	**91.64**	**89.75**

The highest scores are highlighted in bold

1: models with word and character features

2: models with additional domain resource features and linguistic features

3: models with multi-task learning

4: models with large-scale unlabeled datasets

*Indicates that the results are calculated by us according to their reported results in chemical and disease

Comparing group 1 and group 2, we find that rich features indeed improve the performance However, designing and extracting such features is laborious and time-consuming.

While comparing group 1 and group 3, we can see that multi-task learning could improve performance to a certain extent though data augmentation.

Our model and Lee et al. [24] leverage large-scale unlabeled datasets, significantly outperforming other methods. Lee et al. [24] pre-train BioBERT on the datasets with totally 21.3B words, and then fine-tune it on the training data, while our model encoded with BiLSTM is trained on the datasets with only 14.8 M words. The amount of their datasets and the parameter scale of their model are much larger than ours. Even though, our model with vector dimension 100 achieves a competitive performance of Lee et al. [24] with vector dimension 768 on both. This demonstrates the effectiveness of our label re-correction and knowledge distillation strategies. Our weakly labeled dataset is constructed specifically for chemical and disease entity recognition, which is more task-specific than directly using BioBERT. During the training process on the weakly labeled dataset, our word vector is fine-tuned at the same time, so the word vector could remain rich knowledge about chemical and disease entity recognition.

And when we use BioBERT as encoder to re-correct the weakly labeled datasets and train a distilled recognition model, it outperforms Lee et al. [24].

###  Case study

####  Knowledge distillation

**Fig. 4:**
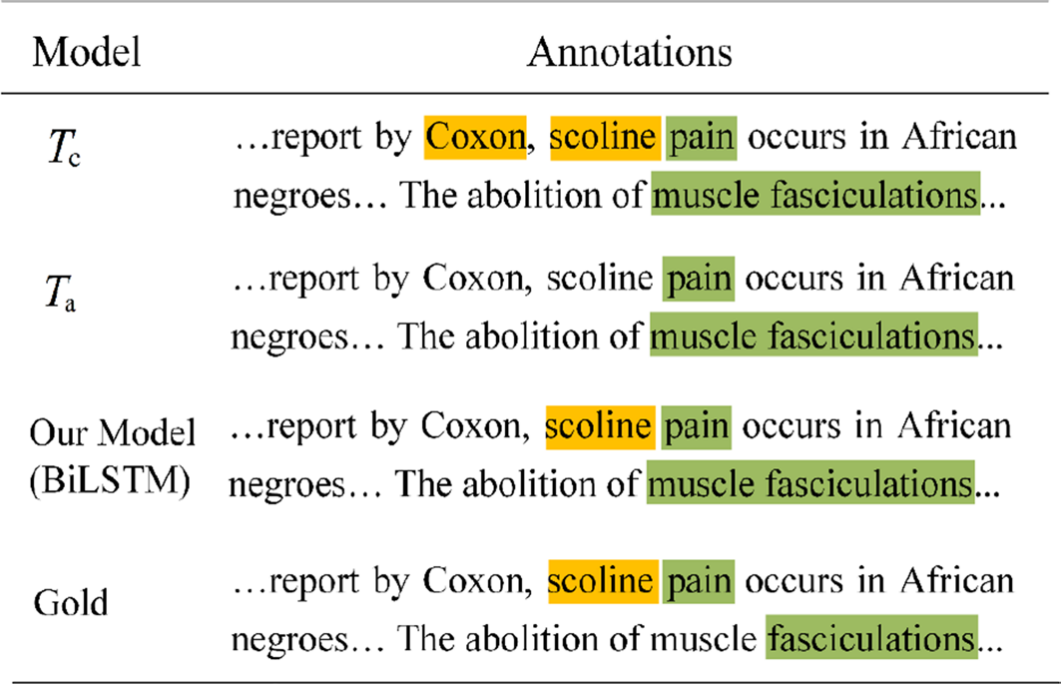
Case study of knowledge distillation effectiveness. Yellow for chemical and green for disease

**Fig. 5:**
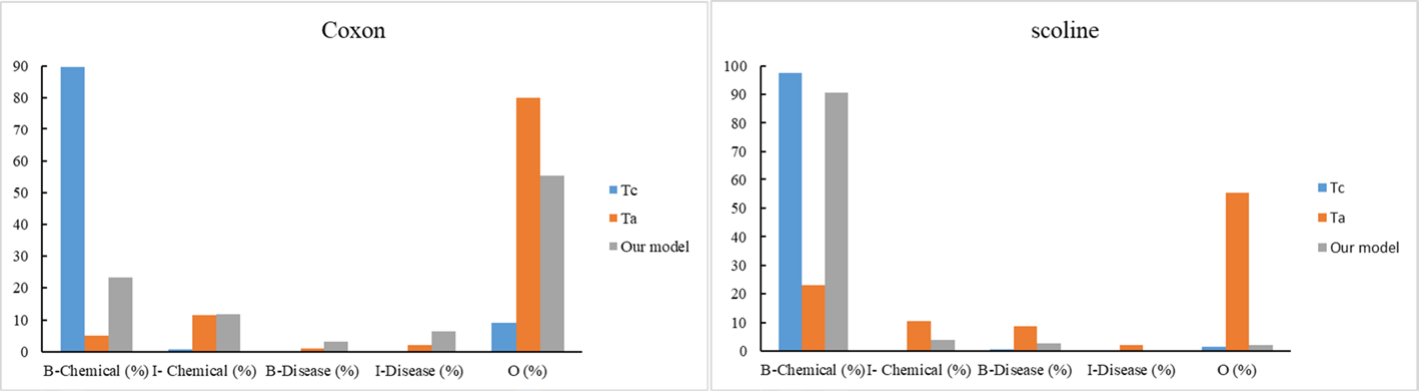
Label probabilities of the words “Coxon” and scoline predicted by $T_{c}$, $T_{{\text{a}}}$ and our model

To better understand in which conditions the knowledge distillation helps, we give the annotations of the same input sentence predicted by the models before and after distillation in Fig. 4. To clearly explain why the student out-performs the teachers, we also output the label probabilities of the words “Coxon” and “scoline” in Fig. 5.

For the word “Coxon”, teacher $T_{{\text{a}}}$ correctly predicts it as “O” with the probability of 80.17%, while teacher $T_{c}$ incorrectly predicts it as “B-Chemical” with the probability of 89.66%. However, through the knowledge distillation, the student selectively learns from the two teachers and balances their probability values. Finally, the probability of label “O” is 55.31%, which is larger than that of label “B-Chemical” with the probability 23.38%. This illustrates that student can effectively distill the trustable knowledge from the teachers.

Similarly, for the word “scoline”, the label probabilities of the two teachers are quite different. The student effectively distills the knowledge from the two teachers, finally assigning the probability of 90.70% to the right label “B-Chemical”.

Finally, we find that the student could identify some synonyms in the CDR. The gold standard annotates “fasciculations” as disease, while our model annotates “muscle fasciculations” as disease. From our understanding, neither our model nor the gold standard are wrong, because the entity our model identified is synonymous with the one in the gold standard.

#### Re-correction

**Fig. 6:**
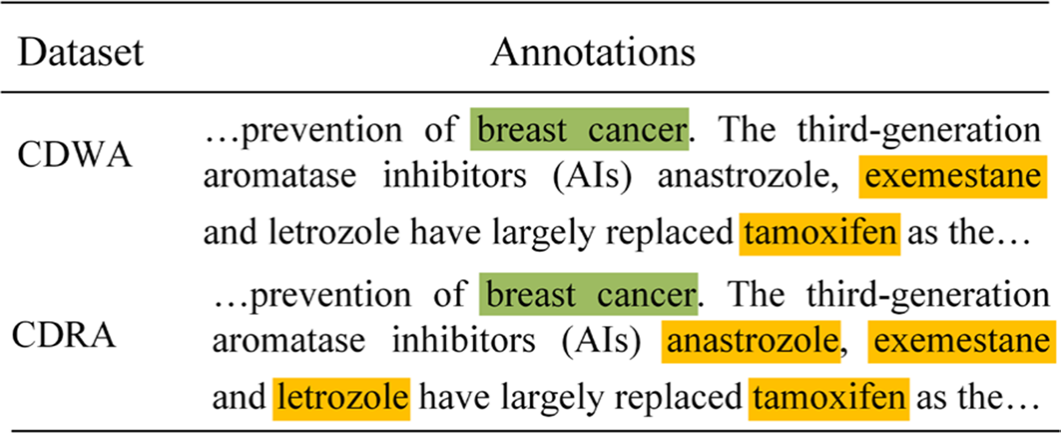
Case study of re-correction effectiveness. Yellow for chemical and green for disease

To better shown that the new corrected dataset is indeed of higher quality than the weakly labeled ones, the annotations of the same input sentence in CDWA and CDRA are shown in Fig. 6.

From the figure, we can see that the missed chemical mention “anastrozole” and “letrozole” in CDWA are got back in CDRA. This proves that the re-correction procedure indeed reduce some false negatives.

## Conclusion

In this paper, we address the problem of insufficient training set that BioNER suffers from. A novel label re-correction strategy is proposed to make full use of PubTator and knowledge bases to obtain two large-scale high-quality datasets for coverage and accuracy, respectively. Further, we introduce knowledge distillation to transfer knowledge from two recognition models into a distilled recognition model. Experiments show that label re-correction benefits recognition significantly and knowledge distillation further improves recognition. As a result, we achieve the new state-of-the-art results on CDR and NCBI Disease. In terms of further work, we would like to integrate semi-supervised learning and multi-task learning to construct large-scale datasets for broader knowledge transfer.

## Data Availability

The datasets generated and code during the current study are available in https://github.com/ZheLiu1996/Label-Re-correction-and-Knowledge-Distillation. The datasets analyzed during the current study are available in the CDR repository, https://biocreative.bioinformatics.udel.edu/tasks/biocreative-v/track-3-cdr/. The datasets analyzed during the current study are available in the NCBI Disease repository, https://www.ncbi.nlm.nih.gov/CBBresearch/Dogan/DISEASE/.
